# Cell-Type-Specific High Throughput Toxicity Testing in Human Midbrain Organoids

**DOI:** 10.3389/fnmol.2021.715054

**Published:** 2021-07-15

**Authors:** Henrik Renner, Katharina J. Becker, Theresa E. Kagermeier, Martha Grabos, Farsam Eliat, Patrick Günther, Hans R. Schöler, Jan M. Bruder

**Affiliations:** ^1^Department for Cell and Developmental Biology, Max Planck Institute for Molecular Biomedicine, Münster, Germany; ^2^Westfälische Wilhelms-Universität Münster, Münster, Germany

**Keywords:** organoids, midbrain, screening, high throughput, automation, toxicity testing

## Abstract

Toxicity testing is a crucial step in the development and approval of chemical compounds for human contact and consumption. However, existing model systems often fall short in their prediction of human toxicity *in vivo* because they may not sufficiently recapitulate human physiology. The complexity of three-dimensional (3D) human organ-like cell culture systems (“organoids”) can generate potentially more relevant models of human physiology and disease, including toxicity predictions. However, so far, the inherent biological heterogeneity and cumbersome generation and analysis of organoids has rendered efficient, unbiased, high throughput evaluation of toxic effects in these systems challenging. Recent advances in both standardization and quantitative fluorescent imaging enabled us to dissect the toxicities of compound exposure to separate cellular subpopulations within human organoids at the single-cell level in a framework that is compatible with high throughput approaches. Screening a library of 84 compounds in standardized human automated midbrain organoids (AMOs) generated from two independent cell lines correctly recognized known nigrostriatal toxicants. This approach further identified the flame retardant 3,3′,5,5′-tetrabromobisphenol A (TBBPA) as a selective toxicant for dopaminergic neurons in the context of human midbrain-like tissues for the first time. Results were verified with high reproducibility in more detailed dose-response experiments. Further, we demonstrate higher sensitivity in 3D AMOs than in 2D cultures to the known neurotoxic effects of the pesticide lindane. Overall, the automated nature of our workflow is freely scalable and demonstrates the feasibility of quantitatively assessing cell-type-specific toxicity in human organoids *in vitro*.

## Introduction

All living things, humans included, are exposed to a plethora of natural and artificial compounds on a daily basis. With progressing industrialization, the number of synthetic compounds in our environment increases. Over the past decades, artificial compound use has permeated many areas of human life: agriculture in the form of fertilizers and pesticides; furniture, electronics, and building materials in the form of flame retardants; clothing and food containers in the form of plasticizers; and, more directly, as drugs for medical treatments. Evaluating the toxicity of such compounds is crucial to ensure human health and safety and is thus an important step in the development and approval of chemical entities (Krewski et al., 2010; Parasuraman, 2011). While many of these compounds have undergone safety testing *in vitro* and in animal experiments *in vivo* (Parasuraman, 2011), their effects often have not been tested in human subjects due to practical and ethical constraints. Hence, in most cases, there is no direct data on the acute or long-term effects on human tissues, and the results obtained from existing model systems may not always reflect the risks to human health (Krewski et al., 2010). For example, even after extensive preclinical development, around 90% of drug candidates fail to pass through clinical trials (Dowden and Munro, 2019) with approximately 20% raising safety concerns (Harrison, 2016; Hwang et al., 2016). Although animal models can generally approximate human physiology and offer the opportunity to study compounds’ impact in entire organisms including the interplay between different organs, species-specific differences remain, and the predictive value of animal models for human toxicity is often unclear (Schmidt et al., 2017; Krewski et al., 2020). Moreover, efficient safety testing of many compounds over a broad range of concentrations requires automated medium- and high throughput workflows, which challenge the capacity, practicality, and ethics of typical animal models (Krewski et al., 2010). Automated *in vitro* models, in contrast, can utilize human cells and may provide opportunities to address species-specific and even tissue-specific toxicities at scale. In particular, well-established protocols based on induced pluripotent stem cells (hiPSCs) (Takahashi et al., 2007) have provided virtually unlimited access to a multitude of human cell types, including those that reflect genotypes and phenotypes from patients with specific diseases (Nirmalanandhan and Sittampalam, 2009; Krewski et al., 2011; Friese et al., 2019; Fritsche et al., 2020). However, these typically two-dimensional (2D) cell culture models may not recreate the specific and diverse cell populations of human tissues *in vivo* with their intricate architectures and interdependent molecular interactions, potentially limiting their predictive value for human toxicity (Bus and Becker, 2009; Hartung and Daston, 2009; Horvath et al., 2016). Moreover, compounds can have distinct effects on specific cell subpopulations of complex tissues *in vivo*, which may not be easily detectable in standard cellular assays that rely on monocultures of single cell lines or cell types (Kasurinen et al., 2018). Thus, traditional 2D toxicity assays, while highly reproducible and well-established, may lack the cell types and molecular mechanisms that lead to toxicities in humans. In order to provide more relevant data during laboratory testing, the evaluation of compound toxicity could benefit from more comprehensive human *in vitro* models that are compatible with high throughput screening approaches. These are designed to better mimic human physiology, including the cellular composition and function of organs, than existing model systems. One promising approach is the use of three-dimensional (3D) cell culture systems, especially in the form of human organ-like microtissues (“organoids”), which provides opportunities to generate more relevant predictions for human toxicity by recreating some of the complexity of human tissues *in vitro* (Nirmalanandhan and Sittampalam, 2009; Krewski et al., 2011; Fatehullah et al., 2016; Truskey, 2018; Takahashi, 2019). The use of advanced human 3D models could be particularly beneficial to neurotoxicity studies, as the human brain differs species-specifically from common animal models including rodents (Schmidt et al., 2017). Furthermore, 3D models may also better recapitulate the native tissue niche of cells (Duval et al., 2017; Ho et al., 2018).

One area of particular interest for toxicity testing is the human midbrain due to its relevance for Parkinson’s disease (PD). PD is the second most common neurodegenerative disease and is marked by loss of dopaminergic neurons in the substantia nigra (Alexander, 2004; Poewe et al., 2017). Approximately 90% of cases are of sporadic and idiopathic origin (Lesage and Brice, 2009; Schulze et al., 2018), and many studies hypothesize that genetic and environmental factors, including exposure to toxicants, may play a role in the long-term etiology of PD (Warner and Schapira, 2003; Sulzer, 2007; Goldman, 2014; Bellou et al., 2016; Tysnes and Storstein, 2017). However, Parkinsonism in humans can also have more direct causes, where specific compounds can selectively and acutely ablate dopaminergic neurons. For example, 1-methyl-4-phenyl-1,2,3,6-tetrahydropyridine (MPTP), a toxic byproduct of drug synthesis, caused PD-like symptoms in illicit drug users that had unintentionally exposed themselves to the compound (Davis et al., 1979; Langston et al., 1983; Goldman, 2014). Following the discovery of MPTP’s effects, similar specific toxicities were identified for other compounds, including the common pesticides rotenone and paraquat (Goldman, 2014). Furthermore, many therapeutic drugs are known to cause drug-induced Parkinsonism (DIP) in patients (the second most common form of Parkinsonism in aged patients after PD) (Shin and Chung, 2012; Lopez-Sendon et al., 2013). While DIP is often reversible as it is mostly caused by the blockage of the dopamine receptor at the postsynapse (Thanvi and Treadwell, 2009; Shin and Chung, 2012; Lopez-Sendon et al., 2013), some drugs, including the antipsychotic haloperidol, may also have additional neurotoxic effects and thus directly damage dopaminergic neurons (Ukai et al., 2004; Mena and de Yebenes, 2006; Nasrallah and Chen, 2017). Human data on the molecular details of these side effects are scarce due to limited access to tissue samples.

Building a midbrain-specific human *in vitro* model for toxicity testing may allow identification of such compounds in the future before they come into widespread contact with humans. While various 3D cell culture models of the brain have already contributed to our understanding of human development and disease (Lancaster et al., 2013; Mariani et al., 2015; Pasca et al., 2015; Jo et al., 2016; Qian et al., 2016; Monzel et al., 2017), it has been challenging to combine their complexity with the high degree of standardization and automation required for high throughput toxicity screens. We have recently developed a highly homogeneous and reproducible 3D model system of the human midbrain (“automated midbrain organoids”, AMOs), which is designed for high throughput screening applications (Renner et al., 2020). With these standardized tools, we can address the effects of compounds on a human tissue-like model system, and, at the same time, on distinct human neural subpopulations, specifically dopaminergic neurons.

To explore whether these organoids can be a useful tool for toxicity testing, we screened a library with 84 unique compounds obtained from the National Institutes of Health (NIH), including pesticides, drugs, flame retardants, and non-toxic controls (see Supplementary Table 1 for a complete list) in AMOs. We then evaluated toxic effects on total cell viability and on dopaminergic neurons. Follow-up dose-response experiments further confirmed the results of the primary screens. Our experiments not only recognized previously known neurotoxicants but also identified a compound with previously unknown nigrostriatal toxicity, 3,3′,5,5′-tetrabromobisphenol A (TBBPA). Finally, our assay detected differences in toxicity values in 2D and 3D cultures, specifically for the known neurotoxic pesticide lindane (Nolan et al., 2012).

Our study highlights the ability to quantitatively measure the toxic effects on both overall viability and specific cellular subpopulations in complex human neural tissues *in vitro*. Additionally, our fully automated workflow facilitates high throughput screening, which is necessary to test a plethora of compounds at different concentrations and in sufficient replicates to allow firm statistical assessment.

## Materials and Methods

### Human Small Molecule Neural Precursor Cell (smNPC) Culture

All cells and organoids were maintained at 5% CO_2_ and 37°C unless noted otherwise. All cell lines in this study tested negative for mycoplasma contamination in sequencing- and PCR-based assays. The human small molecule neural precursor cells (smNPCs) were previously generated and characterized (Reinhardt et al., 2013a) and we performed smNPC culture as described before, with minor modifications (Renner et al., 2020). Briefly, we cultured them on 1% [(v/v) in KO-DMEM/F-12, Thermo Fisher] matrigel (BD Biosciences)-coated 6-well plates (Sarstedt) in N2B27 medium with addition of the small molecules smoothened agonist (SAG, 0.6 μM, Cayman Chemical) and CHIR 99021 (3.6 μM, Axon MedChem, smNPC medium). N2B27 was composed of DMEM/F-12 (Thermo Fisher) and Neurobasal Medium (Thermo Fisher) at a 1:1 ratio, supplemented with 1:400 diluted N2 supplement (Thermo Fisher), and 1:200 diluted B27 supplement without vitamin A (Thermo Fisher), 1% penicillin/streptomycin/glutamine (Thermo Fisher), and 200 μM ascorbic acid (Sigma-Aldrich). Generally, we exchanged the culture medium every second day and split the cells every 5–7 days. We split the smNPCs at a ratio of 1:10–1:20 with accutase (Sigma-Aldrich) treatment for approximately 15 min at 37°C to generate a single-cell suspension. We then transferred the cells to a tube containing DMEM/F-12 with 0.1% bovine serum albumin (BSA, Thermo Fisher) to stop the enzymatic digestion and centrifuged them at 220 g for 2 min. Finally, we resuspended the cell pellet in smNPC medium and seeded them in fresh Matrigel-coated 6-well plates.

### Generation and Maintenance of Automated Midbrain Organoids (AMOs)

We cultured AMOs as previously described with minor modifications (Renner et al., 2020, 2021a). We performed all liquid handling steps (seeding, maintenance, and fixation of organoids) with a Biomek FXP liquid handling station equipped with a 96-channel-pipetting head (Beckman Coulter) and an automated attached Cytomat incubator (Thermo Fisher). To generate 3D aggregates, we digested smNPCs to single cells as described above and seeded them in smNPC medium containing 0.4% (w/v) polyvinyl alcohol (PVA, Sigma-Aldrich) into conical 96-well plates (Thermo Fisher) at a density of 9,000 cells/well. After 2 days, we initiated ventral patterning for 4 days in two feedings by removing CHIR 99021 but maintaining 1 μM SAG, and adding 1 ng/ml brain-derived neurotrophic factor (BDNF, PeproTech) and 1 ng/ml glial cell line-derived neurotrophic factor (GDNF, PeproTech). On day 6, we removed SAG and added 1 ng/ml transforming growth factor beta 3 (TGFβ-3, PeproTech) as well as 100 μM dibutyryl cyclic adenosine monophosphate (dbcAMP, Sigma-Aldrich) to support maturation and midbrain-specific differentiation. We also added a single dose of 5 ng/ml activin A (eBioscience) exclusively on day 6. To further increase the maturation and survival of neuronal cells, we also increased the concentration of BDNF and GDNF to 2 ng/ml each from day 6 onward. All following feedings were done with the same medium as day 6 (except for activin A; after day 6, we refer to this as “standard AMO medium”). In general, the organoid medium was exchanged every second day for the entire duration of culture. The organoids can be maintained for 100 days and longer, depending on the desired degree of maturity. For this study, we used organoids cultured for approx. 50 days post aggregation (as indicated in the Results section). This yielded exactly one AMO per well.

### Primary Organoid Viability Screen

We cultured AMOs for 47 days in our automated system as described above. On day 47, we exchanged the organoid medium to a minimal tox medium (TM, consisting of DMEM/F-12 with 1% N2 supplement and 1% penicillin/streptomycin/glutamine) to reduce possible interference from antioxidants in the B27 media supplement. The next day, we treated the AMOs with the compounds from the library at a concentration of 100 μM in TM with some deviations due to solubility (see Supplementary Table 1 for a complete list of compounds including their final concentrations in the screen). DMSO control samples contained 0.5% (v/v) DMSO, which is equivalent to the amount of solvent in the samples that received compound treatment. After 48 h of incubation with the compounds, we either directly processed the organoids for the 48-h time point or renewed the compounds in TM once to achieve 96 h of incubation. Following either 48 or 96 h of compound incubation, we used the CellTiter-Glo 3D cell viability assay (Promega) to measure the viability of individual organoids, as previously described (Renner et al., 2020). Briefly, the 96-well plates of AMOs and the reagents were brought to room temperature for 30 min. We adjusted the media volume of each well to 55 μl and added an equal volume (55 μl) of the CellTiter-Glo 3D reagent. Next, the samples were shaken on a Thermomixer (Eppendorf) at 900 rpm for 5 min and then incubated for 25 min at room temperature while protected from light. To minimize signal cross-talk between adjacent wells, we transferred the contents of the clear 96-well culture plates to white/opaque 384-well Lumitrac plates (Greiner) with two technical replicates (20 μl each) per sample, after the incubation. We recorded the luminescence immediately after the transfer using a Synergy Mx plate reader (BioTek). We outputted the results to Microsoft Excel for reformatting and then transferred them to GraphPad Prism v9.0.1 (Graphpad Software, Inc.) for plotting and statistical analysis.

In detail: All compounds including their controls were arrayed on a single 96-well plate (see Supplementary Table 1 for well positions), and replicated across three plates for a total of *n* = 3 replicates per condition (except AMO line 2 at 48 h *n* = 2). We also included 4 DMSO controls per plate for a total of *n* = 12 (AMO line 2 at 48 h n_DMSO_ = 8). During our analysis, we first averaged the two technical replicates from the luminescence measurement in Microsoft Excel, yielding one averaged luminescence value for each well of the original 96-well AMO plates. Then, we normalized the luminescence detected in each well to the mean luminescence of the four DMSO controls of the same plate to get a relative organoid viability value. Normalization was performed separately for each plate to account for and correct for potential differences during the luminescence measurements. These plate-normalized viability values were outputted to GraphPad Prism, and we plotted the mean relative viability of all biological replicates ±SEM in Figure 2. We further assessed the homogeneity of the AMOs by calculating the coefficient of variation (CV) for the relative viabilities of the DMSO controls (i.e., we divided the standard deviations by the corresponding means and averaged the resulting CV values for the DMSO controls). To identify compounds that significantly altered the viability of AMOs, we compared the mean relative viability of the treated AMOs with that of the DMSO controls. We used the “analyze” tool in Prism to perform multiple unpaired t-tests, one per compound, assuming Gaussian distribution and the same standard deviation for both populations. We corrected for multiple testing using the Bonferroni-Dunn method and considered results with an adjusted *p*-value < 0.05 as significant.

**Figure 1:**
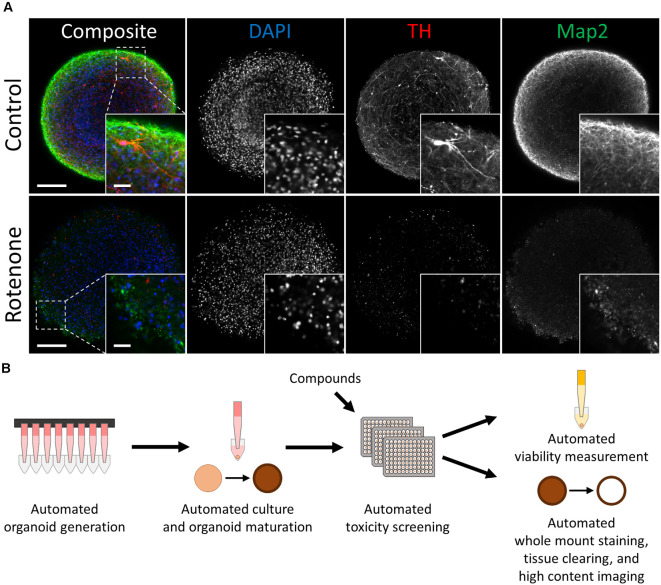
Automated midbrain organoids enable toxicity screening in human tissue-like aggregates. **(A)** Tyrosine hydroxylase (TH) and MAP2 expression declined with exposure to the toxic compound rotenone (bottom) in comparison to control AMOs of the same age (top, both at day 56 of differentiation). The AMO on the bottom was treated with 100 μM rotenone for 48 h starting on day 48 of differentiation and afterward kept under standard culture conditions for six additional days before fixation and whole-mount staining (see “Materials and Methods” section for more details). Representative scanning confocal images of a single optical slice of whole-mount stained and cleared AMOs labeled for the general neuron marker Map2 (green) and dopaminergic neuron marker TH (red). Nuclei were counterstained with DAPI (blue). The inlays in the bottom right corner of each image display enlargements of the sections marked by the dotted squares in the respective composite images. Scale bar = 100 μm (entire AMO), 20 μm (inlays). **(B)** Schematic overview of the high throughput screening workflow for the evaluation of overall and cell type-specific toxicity.

**Figure 2:**
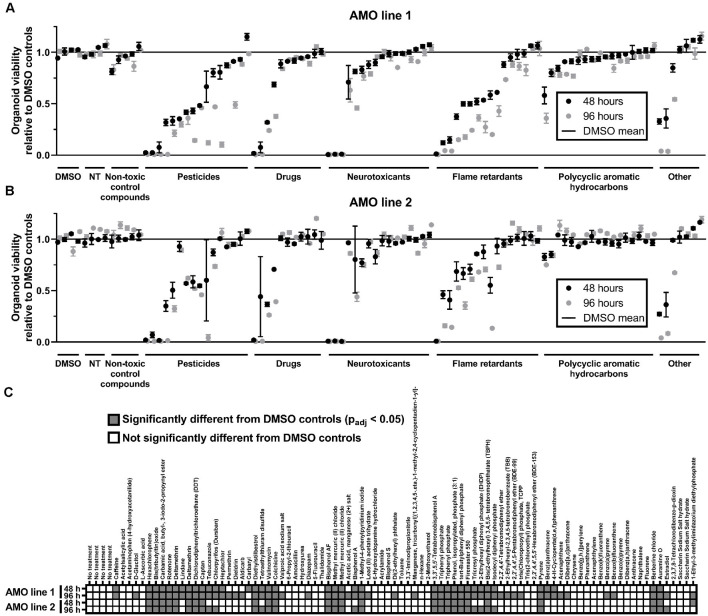
General cell viability identified toxic compounds in an organoid-based high throughput screen. **(A,B)** While toxicant exposure yields a broad range of organoid viabilities, non-treated (NT) and non-toxic controls cluster around 100% of viability relative to DMSO controls. Dot plots depict the mean viability (relative to the DMSO controls) of organoids in the primary screen after 48 (black dots) and 96 h (gray dots) of compound exposure. Compounds are organized by categories listed below the x-axis, then by the viability of AMO line 1 at 48 h within each category. The order of compounds was maintained for subfigures **(A)** (AMOs from cell line 1), **(B)** (AMOs from cell line 2), and **(C)** (statistical analysis) to facilitate comparison. **(C)** Combined heatmap for both AMO lines and time points highlights compounds that significantly changed the AMOs’ viability compared to the DMSO controls. Statistical significance was determined *via* multiple unpaired *t*-tests, and, after correction for multiple testing, we considered *p*_adj_ < 0.05 as significant (see “Materials and Methods” section for details on statistical analysis). *n* = 3 organoids per condition, except n_AMO_ line 2 48 h = 2 and n_AMO_ line 1 48 h chrysene = 2. Plotted is the mean ± SEM. For exact number values see Supplementary Figure 1.

### Whole-Mount Staining and Clearing of Organoids

All processing steps were conducted in an HTS-compatible automated liquid handling system (ALHS, a Biomek FXP liquid handling station). To quantify different cellular subpopulations within the AMOs, we employed our previously described (Renner et al., 2020, 2021b) ALHS-compatible workflow for combined whole-mount staining and clearing, with minor modifications. We fixed the AMOs with 4% PFA (VWR) for 10–15 min and stained the entire aggregates with primary and secondary antibodies for 6 days each. The antibodies used in this study were as follows. Primary: mouse anti-Map2 (1:1,000, Merck Millipore, cat#: MAB3418) and rabbit anti-TH (1:500, Abcam, cat#: ab112). Secondary: donkey anti-mouse IgG, Alexa Fluor 488 conjugated (1:1,000, Thermo Fisher, cat#: A-21202) and donkey anti-rabbit IgG, Alexa Fluor 647 conjugated (1:1,000, Thermo Fisher, cat#: A-31573). The antibodies were diluted in blocking and permeabilization solution consisting of 6% BSA, 0.5% Triton-X 100 (Roth), 0.1% (w/v) sodium azide (Sigma-Aldrich) in PBS (Sigma-Aldrich) and immediately added after fixation following a triple rinse with PBS. We renewed the antibody solutions once after three days. As a nuclear counter stain, we added DAPI (1 μg/ml, Sigma-Aldrich) to the solution containing the secondary antibodies. Between primary and secondary antibody application and after finishing the secondary antibody incubation, we washed the samples overnight with 0.1% Triton X-100 in PBS, with a total of five exchanges of the washing solution. After the whole-mount staining, we performed BABB-based tissue clearing (Dent et al., 1989) to allow image acquisition with single-cell resolution of the entire aggregates. For this purpose, we first dehydrated the AMOs *via* a stepwise methanol (Roth) series (25%, 50%, 70%, 90%, and 100%, each for 15 min). For clearing and imaging, we transferred them to organic solvent-resistant cyclo-olefin 96-well flat-bottom plates (“Screenstar”, Greiner Bio-One). Finally, we incubated the samples for 30 min in 1:1 (v/v) methanol/BABB (BABB consisted of benzyl alcohol (Sigma-Aldrich) and benzyl benzoate (Sigma-Aldrich) at a 1:1 (v/v) ratio) and then kept them in 100% BABB in the dark for storage and subsequent automated high content confocal fluorescent analysis.

### Primary Cell-Type-Specific Toxicity Screen

We prepared and treated the AMOs with the compounds as described for the primary viability screen. On day 50, after 48 h of compound incubation, we replaced the medium with standard AMO medium and maintained the AMOs for 6 additional days under standard culture conditions. This additional time allows the cells killed by the compounds to be cleared from the aggregates. After the 6 days, we fixed the organoids with 4% PFA, whole-mount-stained them for TH and Map2, and tissue-cleared them as described above. To achieve uniform aggregate positioning within the wells before imaging, we tilted the plates off the horizontal plane at 60 degrees for a minimum of 5 min. We performed automated image acquisition of entire 96-well plates on an Operetta high content imager (Perkin Elmer) and analyzed images in the Harmony 4.1 software and/or Columbus version 2.6.0 as previously described (Renner et al., 2020), with adjustments to account for the individual properties of the staining and samples. We acquired three medial planes for each organoid, spaced 36.6 μm apart to avoid double-sampling individual morphological features. For the analysis, we defined the organoid region in each image by summing all channels, removed small localized features with a median filter (10 px), and identified putative organoids/regions of interest (ROIs) with the “find image region” function. To identify *bona fide* AMOs and exclude image artifacts and dust, we selected the ROIs with an area >9,000 μm^2^, roundness >0.4, and mean DAPI intensity >190 arbitrary brightness units (abu). To subtract the general fluorescent background from the TH+ areas in the 647 nm channel, we first ran a Gaussian smoothing filter (10 px) across the 647 nm channel and subtracted the smoothed image from the raw image of the channel to generate a background-corrected (BC) image. We then identified ROIs within the previously selected organoid regions *via* the “find cells” function, algorithm C, and selected them to be TH+ if they were >25 μm^2^ in area and had a brightness between 200 abu and 2,200 abu on the BC image (to exclude potential dust particles, and other miscellaneous image artifacts). For Map2 in the 488 nm channel, we also smoothed the 488 nm channel with a Gaussian smoothing filter (5 px) and subtracted the resulting image from the original. We then ran a sliding parabola algorithm (with a curvature of 10) across the resulting image and again identified Map2+ ROIs within the previously selected organoid regions *via* the “find cells” function, algorithm C on this Map2-BC image and selected them if their area was >10 μm^2^. Finally, the brightness of all selected TH+ (in the raw 647 nm channel) and Map2+ (in the raw 488 nm channel) ROIs was summed for all fields of view of each individual plane, and the data was outputted to Microsoft Excel and GraphPad Prism v9.0.1 for additional annotation, reformatting, plotting, and statistical analysis.

In detail: Our plate setup was identical to the viability screen described above, and we treated four plates per AMO line in parallel, providing *n* = 4 replicates per condition as a starting point. However, as described in the main text, we observed a high amount of sample loss during processing, especially for highly toxic compounds. We thus omitted wells with damaged organoids or no organoids and only included data from wells where our image analysis pipeline could identify a *bona fide* organoid ROI (see above) as well as a signal in both the Map2 488 nm and TH 647 nm channels, to remove other potential imaging artifacts/debris from further analysis (see Supplementary Table 2 for a complete overview of all analyzed sample numbers). The analysis software (Columbus/Harmony) summed the organoid ROIs’ area across all acquired fields of view (FOVs) per well and plane and outputted this number for further analysis. The software also provided the integrated brightness for the respective ROIs identified in the 488 nm and 647 nm channels across all acquired FOVs per well and plane.

**Figure 3:**
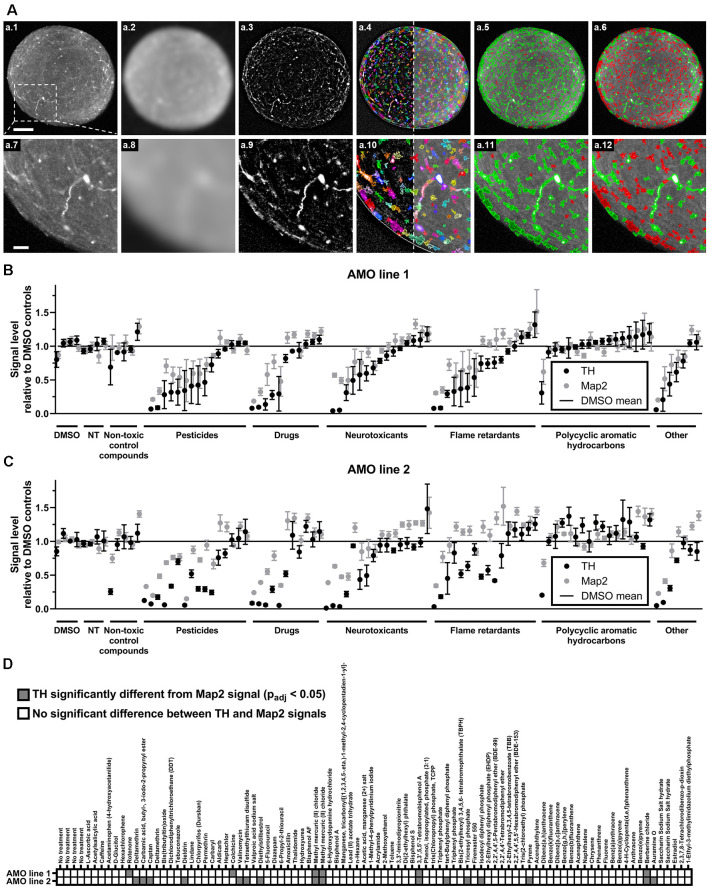
High content image analysis quantified cell-type-specific toxicity in 3D human midbrain-like tissues. **(A)** Outline of the high content image analysis workflow we used to quantify organoid-resident cellular subpopulations for the example of tyrosine hydroxylase (TH)-positive cells. Images represent a single confocal plane through the center of a whole-mount-stained and cleared AMO. The top row **(A.1–A.6)** shows the entire organoid while the bottom row **(A.7–A.12)** contains enlarged sections from the same AMO **(A.1/A.7)**. Raw starting image of the TH channel **(A.2/A.8)**. The TH channel after Gaussian smoothing. **(A.3/A.9)** The TH channel after subtraction of the Gaussian-smoothed image to reduce background (background-corrected channel, BC). **(A.4/A.10)** Putative TH+ regions of interest (ROIs) overlaid on the BC (left of the dotted line) and raw TH channels (right of the dotted line). ROIs were identified on the BC image. **(A.5/A.11)** TH+ ROIs selected based on their size and brightness (green) and rejected ROIs (red). **(A.6/A.12)** Adjustment of the selection criteria to only include the brightest ROIs (green) and discard all others (red). Scale bars: 100 μm **(A.1)**, top, 25 μm **(A.7)**, bottom. **(**B,C**)** Compound treatment induced a wide range of changes within the dopaminergic (TH+, black dots) and general (Map2+, gray dots) neuronal cell populations in AMOs. Data represent mean integrated brightness values of automatically segmented cellular morphologies from confocal slices of whole-mount stained and cleared AMOs. All values were normalized to DMSO controls. In almost all cases, compound exposure resulted in lower TH+ than Map2+ neuronal content. The AMOs and control conditions (i.e., non-treated (NT) and non-toxic control compounds), cluster around 100% of DMSO controls. Compounds are organized by categories listed below the *x*-axis, then by the TH content of AMO line 1 within each category. The order of compounds was maintained for subfigures **(A)** (AMOs from cell line 1), **(B)** (AMOs from cell line 2), and **(C)** to facilitate comparison. **(D)** The combined heatmap for both AMO lines highlights compounds that induced a significant difference between the TH and Map2 content (both relative to DMSO controls) within the AMOs. Statistical significance was determined *via* multiple unpaired *t*-tests, and, after correction for multiple testing, we considered *p*_adj_ < 0.05 as significant (see “Materials and Methods” section for details on statistical analysis). Initially, we started with *n* = 4 organoids per compound. For a complete list of final sample numbers see Supplementary Table 2. Plotted is the mean ± SEM. For exact numeric values, see Supplementary Figure 1.

To generate data per organoid across all planes, we further analyzed data in Microsoft Excel. We first summed the area of the AMOs, the Map2-488 nm intensity, and the TH-647 nm intensity across all planes for each individual well/organoid. We then normalized both Map2 and TH intensity to their respective organoid area. In addition, we normalized the TH and Map2 signals of each well/organoid to the average of the four DMSO controls on the same plate to normalize levels for each individual plate, to ensure technical equivalence across plates. These normalized values were outputted to GraphPad Prism, and we plotted the mean relative signal level of all biological replicates ± SEM in Figure 3. We further utilized the organoid areas to assess the homogeneity of the aggregates’ sizes by calculating the CV for the DMSO controls (i.e., we divided the standard deviations of their areas by the corresponding means and averaged the resulting CV values for the DMSO controls). To identify compounds that induced significant differences between the Map2 and TH content, we compared the mean relative signal levels of TH and Map2 for each compound. We used the “analyze” tool to perform multiple unpaired *t*-tests, one per compound, assuming Gaussian distribution and the same standard deviation for both populations. We corrected for multiple testing using the Bonferroni-Dunn method and considered results with an adjusted *p*-value < 0.05 as significant.

### Dose-Response Analyses

**Figure 4:**
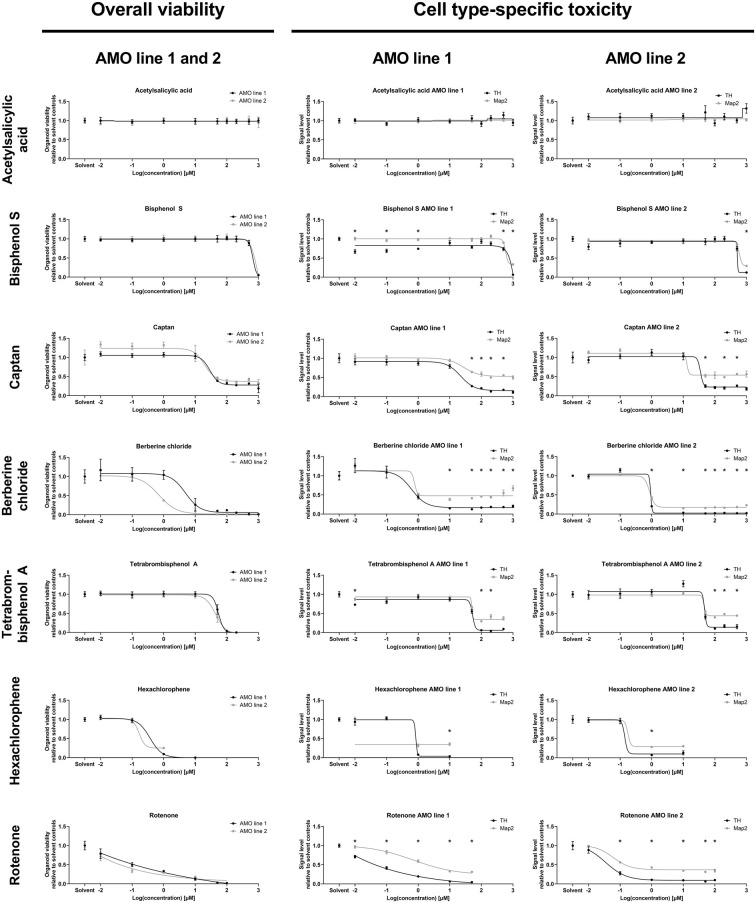
Dose-response analyses of selected compounds confirmed the results of the primary screens. The analysis demonstrates the ability of our organoid-based workflow to detect and quantify typical dose-response behaviors induced by seven representative compounds. The left column lists ATP-based overall AMO viability data; the right two columns show high content imaging-based data for subpopulation-specific TH and Map2 content by cell line. The results are consistent with those of the primary screen at 100 μM concentration (corresponding to “2” on the logarithmic scale) as well as the compound classifications (e.g., ASS as a negative control induces no significant changes at any of the tested concentrations). Depicted are the changes in signal level relative to the DMSO controls plotted against the log base 10 of the compound concentrations in μmol/L. Data points are mean values ±SEM. We initially started with *n* = 8 organoids per condition; for exact sample numbers see Supplementary Table 3. Statistical significance for the difference in Map2 and TH content was determined *via* multiple unpaired *t*-tests, and, after correction for multiple testing, we considered *p*_adj_ < 0.05 as significant (*). For details on curve fitting as well as data and statistical analyses, see the “Materials and Methods” section.

AMOs were cultured as described above for 47 days. On day 47, we exchanged their medium to tox medium (TM), initially without the addition of any compounds. The next day, we replaced the medium with TM containing different concentrations (0.01, 0.1, 1, 10, 50, 100, 200, 500, and 1,000 μM; for rotenone only up to 100 μM due to solubility issues; all in tox medium) of the following compounds: acetylsalicylic acid (Sigma-Aldrich, in DMSO), bisphenol S (Sigma-Aldrich, in ethanol), captan (Sigma-Aldrich, in DMSO), berberine chloride (Sigma-Aldrich, in DMSO), 3,3′,5,5′-tetrabromobisphenol A (Sigma-Aldrich, in ethanol), hexachlorophene (Sigma-Aldrich, in DMSO), and rotenone (Sigma-Aldrich, in DMSO). For each compound, we included *n* = 8 controls per compound treated with the corresponding solvent at an amount equivalent to the highest compound concentration. To assess viability and cell type-specific toxicity in parallel and under the same conditions, we treated all samples with the compounds for 48 h, then exchanged their medium to standard culture medium without compounds and maintained them for an additional 6 days before analysis to allow breakdown and removal of cellular debris. Organoid viability and cell type-specific toxicity were analyzed as described for the primary screens. Here, we acquired five confocal planes spaced 36.6 μm apart in our Operetta high content imager. The image analysis of the AMOs for cell type-specific toxicity assessment required minor adjustments to account for the individual properties of the staining. In short, we selected ROIs to be TH+ if they were larger than 10 μm^2^ in area and had a maximum brightness smaller than 5000 abu. For Map2, we performed Gaussian smoothing with a width of 10 px and omitted the additional sliding parabola filter. The other steps were performed as outlined for the primary cell-type-specific toxicity screen. The data was outputted to Microsoft Excel and GraphPad Prism v9.0.1 for further analysis. In detail: The dose-response series, including the individual solvent controls, was performed for every compound on a separate 96-well plate of AMOs with initially *n* = 8 organoids per concentration. However, as outlined in the main text, especially the highly toxic conditions resulted in a high number of samples being lost during processing (see Supplementary Table 3 for exact sample numbers). Data processing, including normalizations, was performed for each individual compound/plate as outlined above for the primary screens. Significant differences between the Map2 and TH content were also determined in GraphPad Prism as described for the primary screen and with the same parameters (multiple comparisons were performed for the different concentrations instead of compounds). The dose-response curves shown in Figure 4 were fitted using the “nonlinear regression (curve fit)” option, specifically “log(inhibitor) vs. response – Variable slope (four parameters)”, from the “analyze” tool with default parameters, except -2 (log_10_ of 0.01 μM) as minimum *X* value (we used the lowest tested concentration since the log of 0 is undefined for the solvent control condition).

The images of AMOs treated with different concentrations of compounds (Supplementary Figures 3, 4) as well as the images in Figure 1A were acquired on an LSM 700 scanning confocal microscope (Zeiss). For each of the Figures, the acquisition settings for the Map2 and TH channel were kept constant during imaging, the DAPI channel was individually adjusted. The images were outputted to and processed using ImageJ/Fiji (Schindelin et al., 2012). These images were for illustration purposes only and were not used for any measurements or quantifications.

To analyze the relationship between compound concentration and sample loss in Supplementary Figure 5, we averaged the number of remaining samples after compound exposure (see Supplementary Table 3 for a detailed overview) across all seven compounds, both cell lines, and for both readouts (i.e., viability and cell type-specific toxicity) in Microsoft Excel.

We exported the data to GraphPad Prism and directly plotted the average sample numbers for Supplementary Figure 5A. For Supplementary Figure 5B, we emulated a survival curve in GraphPad Prism by creating a “survival data table and graph” with standard settings and parameters. We rounded the average sample numbers to the closest integer and, after rounding, considered every reduction of the sample number by one as an event (“death” in standard survival analyses). Instead of time points, we utilized the different concentrations as x-values with the solvent control at *x* = 0 and every step in concentration increasing *x* by 1. For example, the rounded sample number changed from 8 to 7 at a concentration of 1 μM (the “third” concentration used), thus, we added *x* = 3 as the “time point” for the first event. Finally, we added a “censored” event one step (*x* = 10) after the highest tested concentration to represent the number of samples left at the end of the analyzed concentration range.

### Comparison of the Toxic Effects of Lindane and 6-Hydroxydopamine in 2D and 3D Culture

To compare the toxic effects of compounds on AMOs and 2D-cultured midbrain dopaminergic neurons, we seeded and cultured both in parallel and generated them from the same smNPC starting cell population. For 2D dopaminergic neuron differentiation, smNPCs were seeded in 1% (v/v in KO-DMEM/F-12) Matrigel-coated 6-well plates and standard smNPC medium, following digestion by accutase as described above. From day 2 onwards, they were treated identically with the AMOs (i.e., they received the same media at the same timepoints). To work with the 2D dopaminergic neurons in a screening setting (and have the same plate format as the AMOs), we reseeded them to flat-bottom 96-well plates (“μCLEAR”, black, Greiner) at a density of 70,000 cells/well on day 48. On day 49, the medium of both 2D mDA neurons and 3D AMOs was exchanged to tox medium. The next day, we treated them with different concentrations (0.01, 0.1, 1, 10, 50, 100, 200, 500, and 1,000 μM; controls received the corresponding solvent at an amount equivalent to the highest compound concentration; *n* = 6 per concentration) of the compounds lindane (Sigma-Aldrich, in ethanol) and 6-hydroxydopamine hydrochloride (Sigma Aldrich, in DMSO) in tox medium. After 48 h of incubation, we measured the samples’ viability. For the AMOs, we used the CellTiter-Glo 3D cell viability assay as described above. We measured the 2D mDA neuron viability with the CellTiter-Glo 2.0 cell viability assay (Promega) according to the manufacturer’s instructions. In short, we brought the sample plates and reagents to room temperature for 30 min. We adjusted the media volume of the samples to 50 μl and added an equal volume (50 μl) of the CellTiter-Glo 2.0 reagent. We let the plate shake for 2 min at 900 rpm on a Thermomixer and then incubated them for 10 min at room temperature and protected from light. Immediately afterward, we measured the luminescence on a Synergy Mx plate reader. We outputted the results to Microsoft Excel and GraphPad Prism v9.0.1 for reformatting, plotting, and analysis. Data processing was performed as outlined for the CellTiter-Glo 3D-based viability assay above, except for the averaging of technical replicates, as measurements were performed directly in the culture plate without additional technical replicates.

All other data analysis steps including the statistical analysis to identify significant differences in toxicity between 2D and 3D (*via* multiple unpaired t-tests with the same parameters as above, corrected for using the Bonferroni-Dunn method, and considering *p*_adj_ <0.05 as significant), as well as dose-response curve fitting, were performed as described in detail above. The IC_50_ values were taken from the same regression/analysis as the dose-response curves.

## Results

### General Cell Viability Identified Toxic Compounds in an Organoid-Based High Throughput Screen

Automated midbrain organoids are a highly homogeneous and reproducible 3D cell culture system that mimic relevant features of the human midbrain, including cellular composition, gene expression, and functionality (e.g., dopamine secretion and spontaneous neural activity) (Renner et al., 2020). AMOs possessed a uniform, spherical morphology [coefficient of variation (CV) 4% in size and 3% in viability in DMSO-treated controls]. They also expressed key markers of the human midbrain, most importantly the dopaminergic neuron marker tyrosine hydroxylase (TH) and the more general neuronal marker microtubule-associated protein 2 (Map2; see Figure 1A).

To gain insight into the general/non-cell-type-specific toxicity of each compound, we analyzed the overall viability of individual AMOs from two independent cell lines after either 48 or 96 h of compound exposure based on their ATP content (for the general workflow, see Figure 1B). Overall, exposure to the compound library resulted in a broad range of organoid survival, ranging from close to zero to non-treated (NT) control levels (see Figure 2; for detailed data, see Supplementary Figure 1). In total, 22 of the 84 compounds significantly changed the organoids’ viability compared to the DMSO controls in both cell lines and at both time points, with 96 h of exposure increasing the number of significant results (see Figure 2C). Raising the exposure time from 48 to 96 h further decreased the viability for 71 (AMO line 1) or 41 (AMO line 2) out of the 84 compounds (see Figures 2A,B, Supplementary Figure 1). Interestingly, the average viability differences between the two exposure times varied by compound class, i.e., flame retardants and pesticides reduced viability by approximately an additional 15% between 48 and 96 h of exposure, drugs, and neurotoxicants by approximately 5%. Finally, the correlation between different replicates/plates from the same AMO line was higher (*R*^2^ ≥ 0.96; see Supplementary Figures 2A,B) than between AMOs derived from different cell lines (*R*^2^ = 0.84; see Supplementary Figure 2C). Generally, organoids derived from cell line 2 survived exposure to many compounds better than those from cell line 1 (see Supplementary Figure 2C). Overall, viability evaluation provides a fast and highly reproducible method of detecting toxicity in human midbrain-like tissues.

### High Content Image Analysis Quantified Cell-Type-Specific Toxicity in 3D Human Midbrain-Like Tissues

One of the advantages of organoids over traditional 2D cell culture systems is their ability to maintain multiple organ-specific cell types in a 3D cellular architecture resembling their *in vivo* niche (Lancaster and Knoblich, 2014; Duval et al., 2017; Ho et al., 2018). While the measurement of the organoids’ overall viability provides general insight into the toxicity of different compounds, it does not resolve information about cell type-specific effects. Despite their prevalent role in PD and induced Parkinsonism, midbrain dopaminergic (mDA) neurons are only a select subpopulation of the midbrain and our AMOs, which *in vivo* selectively atrophy prior to other cell types. Thus, it would be advantageous to quantify mDA neuron toxicity separately from general AMO toxicity. We selectively quantified the dopaminergic neurons within AMOs (marked by TH) and the general neuronal population (marked by Map2) via automated high content image analysis after whole-mount staining and tissue clearing. Our automated image analysis pipeline enables the unbiased quantification of entire 96-well plates of whole organoids down to the level of single cells (see Figure 3A for an illustration and outline of the high content acquisition and image analysis; a detailed description can be found in the “Materials and Methods” section).

While almost all compounds decreased the level of TH-positive mDA neurons more strongly than the Map2-positive overall neurons (see Figures 3B,C; and Supplementary Figure 1 for numerical values), 17 compounds resulted in a statistically significant difference in reduction between TH and Map2 (Figure 3D). Of those 17, 12 were unique to AMO line 2 and three compounds were shared between both lines: the organometallic compound methyl mercuric (II) chloride, the brominated flame retardant 3,3′,5,5′-tetrabromobisphenol A (TBBPA), and the isoquinoline alkaloid berberine chloride (BC; see Figure 3D). As methylmercury has previously been described to impair dopaminergic function and to have similar neurotoxic effects to the well-known nigrostriatal toxicant 1-methyl-4-phenylpyridinium (MPP+, the active metabolite of MPTP) (Dreiem et al., 2009; Shao et al., 2015; Tiernan et al., 2015), we decided to focus on TBBPA and berberine chloride and further analyzed them as well as five other compounds of interest in dose-response experiments.

### Dose-Response Analyses of Selected Compounds Confirmed the Results of the Primary Screens

We followed up the primary screens with dose-response studies exploring the relationship of compound exposure and their toxicity across five orders of magnitude (0.01 μM–1,000 μM) with higher sample numbers. Again, we separately assessed both mDA neuron-specific toxicity (*via* TH abundance) and general neuronal content (*via* Map2 abundance) at the protein level, and the overall viability *via* an ATP-based lytic biochemical assay. We selected five compounds in addition to BC and TBBPA, a negative (non-toxic) control compound, acetylsalicylic acid (ASS), and a compound with intermediate toxicity (captan). We included Bisphenol S (BPS) as a compound with published neurotoxic effects (Qiu et al., 2019) that displayed no toxicity in our primary screens. We also included the highly toxic pesticide hexachlorophene (HCP) and rotenone as a well-published toxic control (see Supplementary Figures 3, 4 for illustrations of the changes induced by compound treatment *via* representative confocal TH and Map2 images). The selected compounds had very similar effects on the two different cell lines as in the primary screens (also see Supplementary Figure 2C), and variances between cell lines remained low for both viability and subpopulation-specific effects in our dose-response studies (Figure 4).

Generally, the dose-response experiments corroborated findings from earlier experiments. ASS remained non-toxic across all tested concentrations. BC, HCP, Rotenone, and TBBPA strongly reduced viability, MAP2, and TH content. In general, we noticed a loss of samples under highly toxic conditions (see Supplementary Figure 5, and for analyzed sample numbers, see Supplementary Table 3), as a consequence of the compounds’ toxicity (also see “Discussion” section). The effects of captan were intermediate compared to ASS and the more toxic compounds, as expected. BPS was toxic at the highest concentrations tested (≥500 μM) and had no discernible effects at the concentration used in the primary screens (100 μM). Compound exposure reduced TH levels more than Map2 levels, except in ASS, which had no impact on the protein levels across all doses. Captan, BC, TBBPA, and rotenone significantly reduced TH levels compared to Map2 over a broad range of concentrations. HCP and BPS showed similar significant differences only at the highest toxic concentrations, confirming the validity of the data from the primary screens.

### The Pesticide Lindane is Significantly More Toxic in Automated Midbrain Organoids Than 2D-Cultured Dopaminergic Neurons

**Figure 5:**
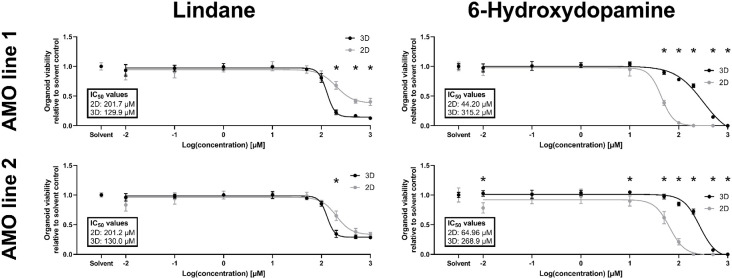
The pesticide lindane is significantly more toxic in automated midbrain organoids than 2D-cultured dopaminergic neurons. While the well-established toxicant 6-hydroxydopamine (6OHDA, right column) was significantly more toxic in 2D (gray symbols) than 3D (black symbols), the toxicity of the pesticide lindane (left column) was significantly higher in 3D AMOs than 2D midbrain dopaminergic (mDA) neurons. We generated AMOs and mDA neurons from the same starting cell lines and batches, cultured them in parallel, and treated them with the compounds for 48 h starting on day 50 before measuring their viability. Data show viability relative to their corresponding solvent controls plotted against the log base 10 of the compound concentrations in μmol/L. Graphs depict mean values ±SEM with *n* = 6 organoids per condition. Statistically significant differences between the 2D and 3D culture formats were determined *via* multiple unpaired *t*-tests, after correction for multiple testing, we considered *p*_adj_ < 0.05 as significant (*). For details on curve fitting, IC50 calculations, as well as data and statistical analysis, see the “Materials and Methods” section.

Despite constant progress in the field of organoids, there is limited data on whether 3D *in vitro* models better recapitulate human toxicity *in vivo* than existing 2D models. In humans, the pesticide lindane has neurotoxic effects, and exposure in some cases has resulted in death (Nolan et al., 2012). However, a previous study with 2D cultured dopaminergic neurons reported only mild toxicity (Sharma et al., 2010). Our primary screening data indicated strong possible toxicity in AMOs. Thus, we decided to further investigate the toxic effects of lindane in our AMOs and compare them to 2D dopaminergic neurons from the same cell lines and cultured for the same amount of time. In detailed dose-response studies, lindane proved to be more toxic than the well-published mDA neuron-specific toxicant 6-hydroxydopamine (6OHDA), which we had previously used as positive toxic control in AMOs (Renner et al., 2020). While 6OHDA treatment had a significantly stronger toxic effect in 2D than 3D (see Figure 5, AMO line 1 IC_50_ values: 2D = 44.20 μM, 3D = 315.2 μM; AMO line 2: 2D = 64.96 μM, 3D = 268.9 μM), lindane was significantly more toxic in AMOs than in the 2D-cultured dopaminergic neurons (see Figure 5, AMO line 1 IC_50_ values: 2D = 201.7 μM, 3D = 129.9 μM; AMO line 2: 2D = 201.2 μM, 3D = 130.0 μM). As expected from the primary screen, AMO line 1 was more severely affected by lindane with a higher discrepancy between 2D and 3D cultures than AMO line 2 (see Figure 5, AMO line 1 showed significant differences for concentrations ≥200 μM, AMO line 2 only at 200 μM and absolute viability levels in 3D). These results may indicate differences in the potential of 3D and 2D cultures to provide predictions for human toxicity.

## Discussion

Here, we utilized an automated organoid model of the human midbrain to screen for the general neurotoxic and dopaminergic neuron-specific toxic effects of a library of 84 compounds as a proof-of-principle for high throughput campaigns. In our hands, the throughput of the workflow is currently only limited by the available equipment. Liquid handling steps for organoid generation and media exchanges were below one minute per 96 samples, despite not having been optimized for speed. The current bottleneck of the workflow is imaging, as our current first-generation imager acquired one 96-well plate of entire whole-mount-stained and cleared AMOs in approximately one day (ca. 12,000 images per plate). However, similar but newer and more advanced microscopes can acquire the same amount of imaging data in a fraction of the time (potentially down to approximately 1 h per 96-well plate with similar acquisition settings) and, thus, drastically increase the throughput of the entire workflow. Reducing the number of imaging planes or channels can further reduce acquisition times. Here, we strove to provide a comprehensive proof-of-principle analysis workflow for toxicity evaluation in 3D. Many steps can be further optimized for speed.

To our knowledge, the work presented here is the first example of an organoid-based screen that simultaneously evaluates the effect of compounds on viability and cellular composition, as well as the first screen to employ midbrain organoids. Other studies previously employed 3D neural aggregates for toxicity testing (Moors et al., 2009), including one example of a screening-compatible setup (Sirenko et al., 2019). However, these aggregates had a cortical-like neural phenotype and did not display the same extent of self-organization and tissue-like cellular architecture as AMOs. Existing high throughput-compatible readouts focus on bulk analyses, including viability (Sirenko et al., 2019), and do not provide information about the subtype-specific effects of toxicants on the cellular composition.

In contrast to these and other existing model systems for toxicity testing, our organoid workflow offers several distinct advantages. AMOs contain multiple, self-organized organ-specific cell types, and their interplay in a 3D human tissue-like environment has the potential to offer more physiologically relevant results than standard 2D monocultures or simpler aggregates. In addition, the combined analysis of organoid viability and specific neuronal subpopulations enables the quantification of overall toxicity and specific cell types, as demonstrated here for TH+ dopaminergic and Map2+ general neurons. Our approach is easily adaptable; the use of a different set of markers directly enables the quantification of other cell types (e.g., astrocytes or neural precursor cells) without requiring any other additional changes to the system.

While AMOs and organoids, in general, are promising tools, major challenges remain. Generally, brain organoids only reach states of cellular maturity similar to early- to mid-fetal development (Camp et al., 2015; Amiri et al., 2018) or require extended periods of culture (approximately 250–300 days) to resemble postnatal stages (Gordon et al., 2021). This may also have implications for the modeling of diseases that typically appear late in life, including PD. It is likely that organoid-based PD models will have to artificially age the organoids to elicit phenotypes more representative of the human disease [similar to existing 2D-based iPSC-derived PD models, which e.g., use additional external stressors to induce more severe phenotypes (Reinhardt et al., 2013b)]. Moreover, brain organoids’ lack of functional immune and vascular systems including a blood brain barrier (BBB) restricts their potential to react physiologically to various types of challenges, such as cellular insults or disease. Therefore, current efforts try to combine the missing elements to create more complex organoid systems, which include the cell types and physiologies required to more closely mimic mature human neural tissues (Abud et al., 2017; Bagley et al., 2017; Lin et al., 2018; Pham et al., 2018; Kim et al., 2020; Shi et al., 2020). Whether these next-generation organoids will be amendable to automation as well as homogeneous and reproducible enough for screening applications will determine their utility as tools for medium- and high throughput scenarios. With their current shortcomings, organoid-based approaches are not advanced enough to replace the complex physiologies of animal testing. However, compounds that show strong toxicities in early *in vitro* trials including organoids can be eliminated from further downstream animal testing, thus reducing the number of compounds in developmental pipelines that undergo animal-based toxicity evaluation.

Screening-compatible strategies that are scalable for very large amounts of samples require simple, robust readouts with low variances. Chemical viability assays, including the lytic ATP-based assay we employed in our primary screens, are straightforward, cost-effective, and yield low variances (see Figure 2, CV for viability 3.1% for DMSO controls). This attests to the high degree of reproducibility attained in our system. While imaging-based assays including whole-mount immunostaining enable quantification of multiple cell populations at the single-cell level, their extra processing steps require additional time, resources, and handling and result in higher variances. Consequently, the variances in our immunostaining-based primary screen exceeded those in our chemical viability data (see Figure 3, CV for TH and Map2 11.4% for DMSO controls). However, higher sample numbers can increase the confidence in mean values, and compensate for the additional variance of the immunostaining-based analysis (see dose-response data, Figure 4).

Interestingly, all 17 compounds with significant cell-specific mDA neuron toxicity were also toxic in the general viability screen. For future identification of midbrain-specific toxicity in larger screens, it may be advantageous to run two tiers of nested screens: one primary screen focusing on general bulk cell survival, the hits of which would undergo further mDA neuron-specific toxicity testing in a secondary screen. This avoids spending resources to test harmless compounds and allows increasing replicates for the most relevant/toxic entities in secondary screens.

During our dose-response experiments, we noticed a loss of samples that correlated with escalating doses of toxic compounds (see Supplementary Figure 5). We observed similar trends for the primary cell type-specific toxicity screen, where low viability in the chemical screen correlated with sample loss in the protein-based readouts (see Supplementary Figure 1, Supplementary Table 2). Under non-toxic control conditions, we have a sample retention of over 90% (Renner et al., 2020), thus we consider the propensity for the absence of samples under toxic conditions as an indirect readout akin to a survival analysis (see Supplementary Figure 5B).

The high reproducibility of our setup identified cell lines as an important factor governing response to a wide array of external toxic stimuli. While the correlation between different plates/replicates from the same AMO line was high, there were distinct differences between the two lines (see Supplementary Figure 2). In particular, we observed that AMO line 2’s viability was generally less vulnerable to the compound treatment than line 1’s, including fewer statistically significant toxic results (see Figure 2, Supplementary Figure 2). Various studies have addressed both genetic and non-genetic differences between different pluripotent stem cell (PSC) lines, which can also influence their differentiation capacities and drug responses (Allegrucci and Young, 2007; Pekkanen-Mattila et al., 2009; Matsa et al., 2016; Carcamo-Orive et al., 2017; Sun et al., 2018; Volpato and Webber, 2020). As the smNPC cell lines we used to generate AMOs originate from PSCs (Reinhardt et al., 2013a) from distinct donors, differences in their drug responses were expected. While the causal link is not clear in all cases, diverging responses of PSC-derived cells to compounds might be indicative of the donors’/patients’ reactions to them (Matsa et al., 2016). Overall, our results are consistent with previous studies demonstrating cell line-dependent variances and further highlight the importance of utilizing multiple lines in testing compounds relevant for human health to account for individual differences.

Across both AMO lines, we identified three compounds with significant dopaminergic neuron-specific toxicity in our primary screen. The organometallic compound methyl mercuric (II) chloride, the brominated flame retardant TBBPA, and the isoquinoline alkaloid berberine chloride (see Figure 3D). While methylmercury has been described to impair dopaminergic function (Dreiem et al., 2009; Shao et al., 2015; Tiernan et al., 2015), we are not aware of any previous studies directly implicating TBBPA with dopaminergic neuron toxicity. However, another brominated flame retardant (1,2,5,6,9,10-hexabromocyclododecane, HBCDD), impairs the dopaminergic system (Genskow et al., 2015). For berberine, previous reports are inconclusive. Some studies reported positive and neuroprotective effects of berberine treatment in chemically-induced *in vivo* PD models (Kim et al., 2014; Negahdar et al., 2015; Wang et al., 2021), while others describe neurotoxic effects including damage to dopaminergic neurons (Kwon et al., 2010; Shin et al., 2013). Our results clearly support the previous studies indicating neurotoxicity and degeneration of dopaminergic neurons (Kwon et al., 2010; Shin et al., 2013). For TBBPA, we are only aware of a single study investigating the consequences of TBBPA exposure on humans, which measured the effect of low-level exposure on neurobehavioral function in adolescents and did not show a significant influence (Kicinski et al., 2012). While extrapolating from AMOs to *in vivo* effects in humans is tenuous, our results suggest using additional caution when encountering TBBPA.

Finally, we compared the toxicity of the pesticide lindane between 2D and 3D cultures. A previous study with 2D-cultured dopaminergic neurons reported only mild toxicity for lindane alone (Sharma et al., 2010) while there have been numerous accounts of neurotoxic and other adverse effects, in some cases even resulting in death, for lindane in humans (Nolan et al., 2012). In 2D, adherent cells are directly exposed to the toxicant, whereas in 3D, the compound may have to diffuse through a number of cell layers before reaching cells located in the core of an AMO. Therefore, a given set of cells should be more susceptible to toxic effects of a compound in 2D culture than in 3D culture under otherwise equal conditions (i.e., the same concentrations and incubation times). Treatment with 6OHDA, a well-established mDA neurotoxicant, confirmed this notion (see Figure 5). However, lindane was significantly more toxic and had a lower IC50 value in 3D AMOs than 2D midbrain dopaminergic neurons across both tested cell lines (see Figure 5). In combination with the human toxicity reported for lindane, these results indicate intriguing differences in the abilities of 3D and 2D model systems to provide predictions for human toxicity. However, more detailed analyses, e.g., into the underlying molecular mechanisms behind this difference, are required to comprehensively evaluate whether the results from 3D model systems like AMOs are indeed more predictive for human neurotoxicity than those from standard 2D models.

In conclusion, we were able to show that toxicity screening in human midbrain organoids is not only feasible but, in some cases, also yields different estimations of human toxicity than existing 2D cell culture systems. Moreover, we were able to demonstrate the utility of quantitatively assessing cell-type-specific toxicity to identify compounds with potential nigrostriatal toxicity for humans, including TBBPA, which had previously not been implicated with adverse effects on the midbrain. Future studies could utilize AMOs derived from iPSCs of both, sporadic and hereditary PD patients, as well as healthy control or gene-corrected cells. These disease models could help further explore possible links between environmental toxicants and PD, or they could help screen novel therapeutics that help boost mDA neuron survival in a 3D human midbrain tissue-surrogate.

While we tested a library of mostly known neurotoxicants for this proof-of-principle, the entire workflow presented here is fully automated and freely scalable. It can, thus, directly be applied to other screening campaigns with different target cell populations, including larger libraries and toxicity testing of novel compounds.

## Data Availability Statement

The original contributions presented in the study are included in the article/Supplementary Material, further inquiries can be directed to the corresponding author/s.

## Author Contributions

HR designed and carried out experiments, interpreted results, and wrote the manuscript. KB contributed to the primary screens and dose-response experiments. TK and MG contributed to organoid culture and the primary screens. FE contributed to the dose-response experiments. PG contributed to the comparison of 2D and 3D toxicity. HS contributed to the conception of the project and interpretation of results. JB contributed to the high content image analysis, conception of the project, data review, interpretation of results, and wrote the manuscript. All authors contributed to the article and approved the submitted version.

## Conflict of Interest

The AMO generation and analysis workflow is the subject of the patent application EP 18 19 2698.0-1120 to the European Patent Office, where HR, MG, HS, and JB are inventors. The remaining authors declare that the research was conducted in the absence of any commercial or financial relationships that could be construed as a potential conflict of interest.
