# Supplementary material for: Cell-Type-Specific High Throughput Toxicity Testing in Human Midbrain Organoids
Source: Front Mol Neurosci. 2021 Jul 15;14:715054. doi: 10.3389/fnmol.2021.715054 (PMC8321240; doi:10.3389/fnmol.2021.715054)
Supplement: Supplementary file 1 [file Data_Sheet_1.docx]

Supplementary Material

**Supplementary Figures**

**

**

**Supplementary Figure 1. Compound exposure has consistent effects on AMOs across two independent cell lines, both on the viability and on the presence of neuronal subpopulations**

The color heatmap, including a comprehensive overview of the primary screens’ results for both viability and protein-based methods (i.e. cell type-specific toxicity), displays broad parallel trends of toxicity between the different cell lines and assays. Numbers represent mean values per condition relative to the DMSO controls (See methods section for detailed information on data analysis). Sample numbers for the viability data: n = 3 organoids per compound, except n_AMO line 2 48 h_ = 2 and n_AMO line 1 48 h chrysene_ = 2. For the TH and Map2 analysis, we started with n = 4 organoids per condition (See Supplementary Table 2 for final sample numbers; for blank cells, no value could be calculated due to missing samples). PAH = Polycyclic aromatic hydrocarbons.





**Supplementary Figure 2. The viability of compound-treated organoids displayed a higher correlation within than between cell lines**

**a/b)** Dot plots of the viability of individual organoids (relative to their respective DMSO controls) after 96 hours of compound treatment. All compounds including their controls were arrayed on a single 96-well plate, and replicated across 3 plates for a total of n = 3 replicates per condition (See Supplementary Table 1 for well positions). Here, we compared reproducibility across different replicates/plates of the same condition by plotting pairwise combinations of identically treated plates. For both AMO line 1 (a) and 2 (b) the correlations between the individual plates/replicates were above R^2^ = 0.96 for all comparisons. **c)** Correlates the corresponding average viabilities for each compound relative to DMSO controls across organoids of the two cell lines (n = 3 replicates for each data point). The variance between cell lines with R^2^ = 0.84 was higher than between replicates of the same cell line. Overall, AMO line 2 had a higher viability after exposure to the compounds than AMO line 1. In a-c, the black line represents a perfect correlation between replicates. Compounds selected for dose-response experiments (see Figure 4) are marked in red. ASS = Acetylsalicylic acid, BPS = Bisphenol S, BC = Berberine chloride, HCP = Hexachlorophene, TBBPA = 3,3’,5,5’-tetrabromobisphenol A. **d)** Heatmaps of the mean organoid viability (n = 3 replicates, relative to DMSO controls, reflecting experimental layouts of compounds on the screening plates) after 96 hours of compound treatment for AMO line 1 (left) and 2 (right). Location of compounds on the plate did not result in any obvious positional biases. Positions of DMSO controls are indicated by yellow boxes.

**
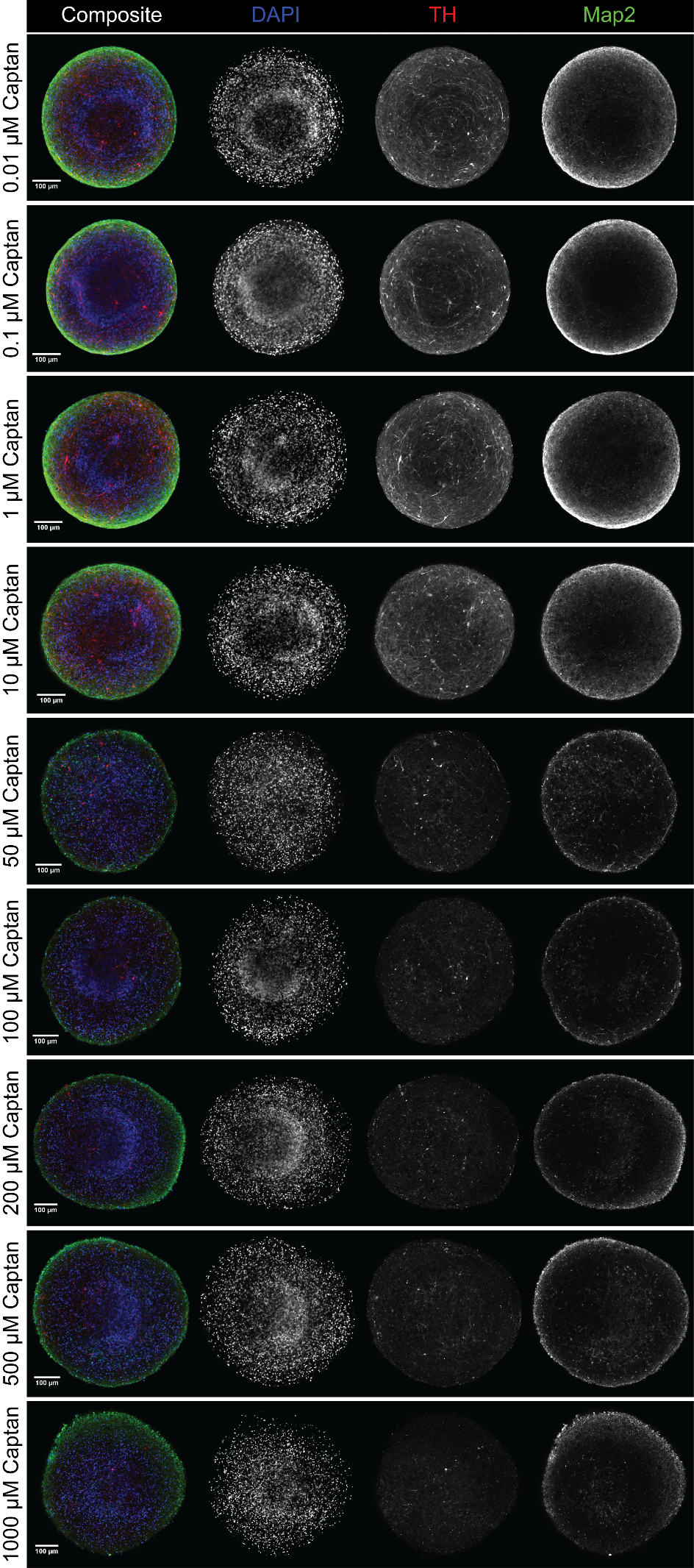
**

**Supplementary Figure 3. Captan reduced dopaminergic and general neuronal content in AMOs**

Increasing concentrations (especially above 10 μM) of captan reduced TH and Map2 signal in AMOs. Scanning confocal images of a single medial optical slice of whole mount-stained and cleared AMOs showing the presence of the general neuron marker Map2 (green) and dopaminergic neuron marker TH (red), with nuclei counterstained with DAPI (blue). AMOs were treated for 48 hours starting on day 48 of differentiation and afterwards kept under standard culture conditions for six additional days before fixation and whole mount staining; the image acquisition settings for the Map2 and TH channels were identical between different AMOs (See methods section for details).


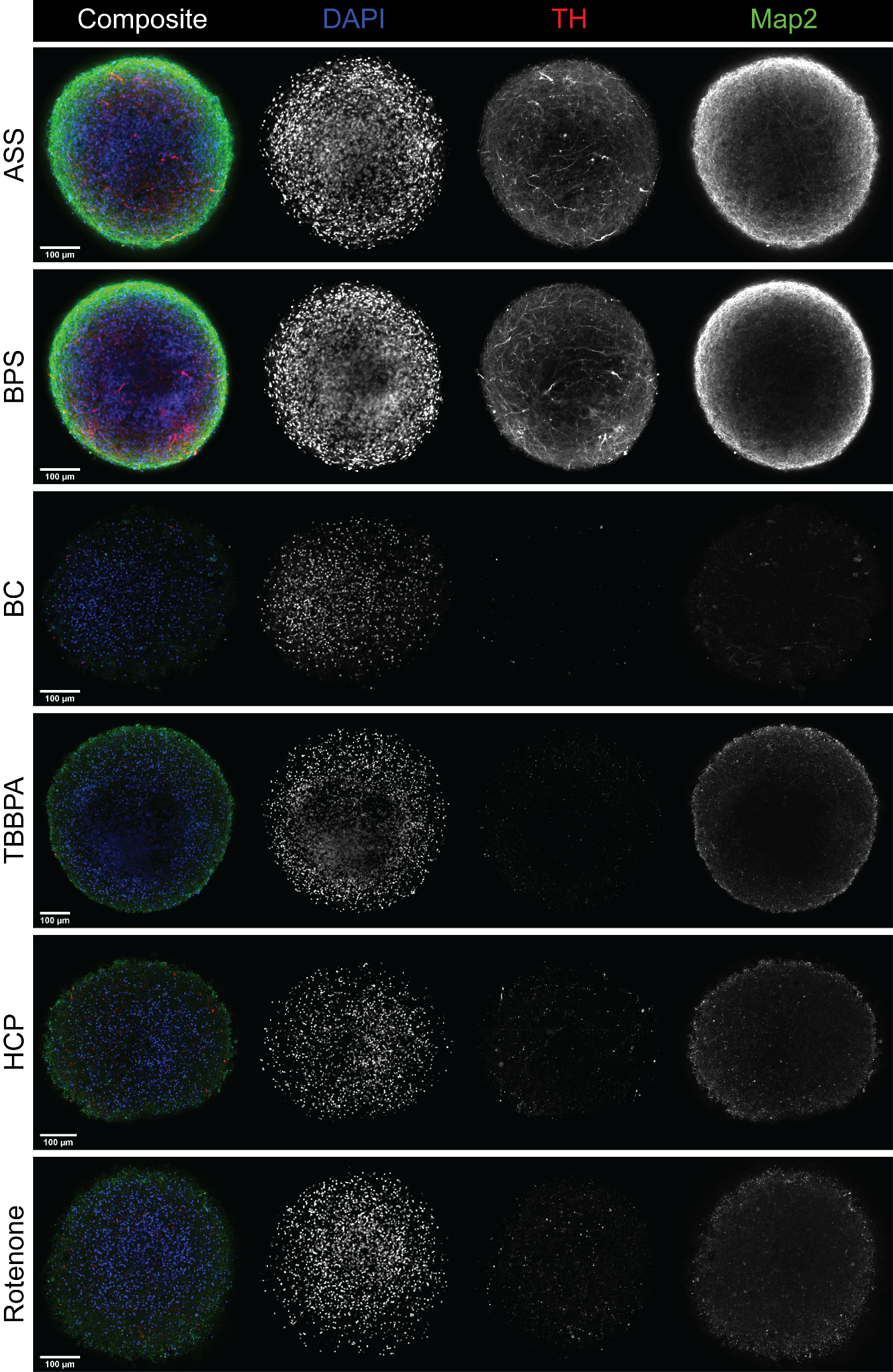


**Supplementary Figure 4. Cellular abundance and morphology of Map2 and TH confirmed prior data in high content dose-response experiments**

Representative scanning confocal images of single medial optical slices of whole mount-stained and cleared compound-treated AMOs reflected general neuronal content (Map2, green) and dopaminergic neuron content (TH, red) consistent with the compounds’ classification (e.g. rotenone as a highly- and ASS as a non-toxic control). Samples originated from the high content dose-response studies and received a medium dose of the compounds (100 μM) to facilitate discerning the compounds’ effects on the presence and morphology of the key markers Map2 and TH. Since toxicity correlated with sample loss (For details, see Table S3 and Figure S5), HCP is shown at 10 μM, the highest concentration retaining samples. AMOs received compound treatment for 48 hours starting on day 48 of differentiation and were afterwards kept under standard culture conditions for six additional days before fixation and whole mount staining; the image acquisition settings for the Map2 and TH channels were identical between different AMOs (See methods section for details). For an example of the dose-dependent effects of compound treatment on TH and Map2, please refer to the data for captan in Figure S3. ASS = Acetylsalicylic acid, BPS = Bisphenol S, BC = Berberine, HCP = Hexachlorophene, TBBPA = 3,3’,5,5’-tetrabromobisphenol A.


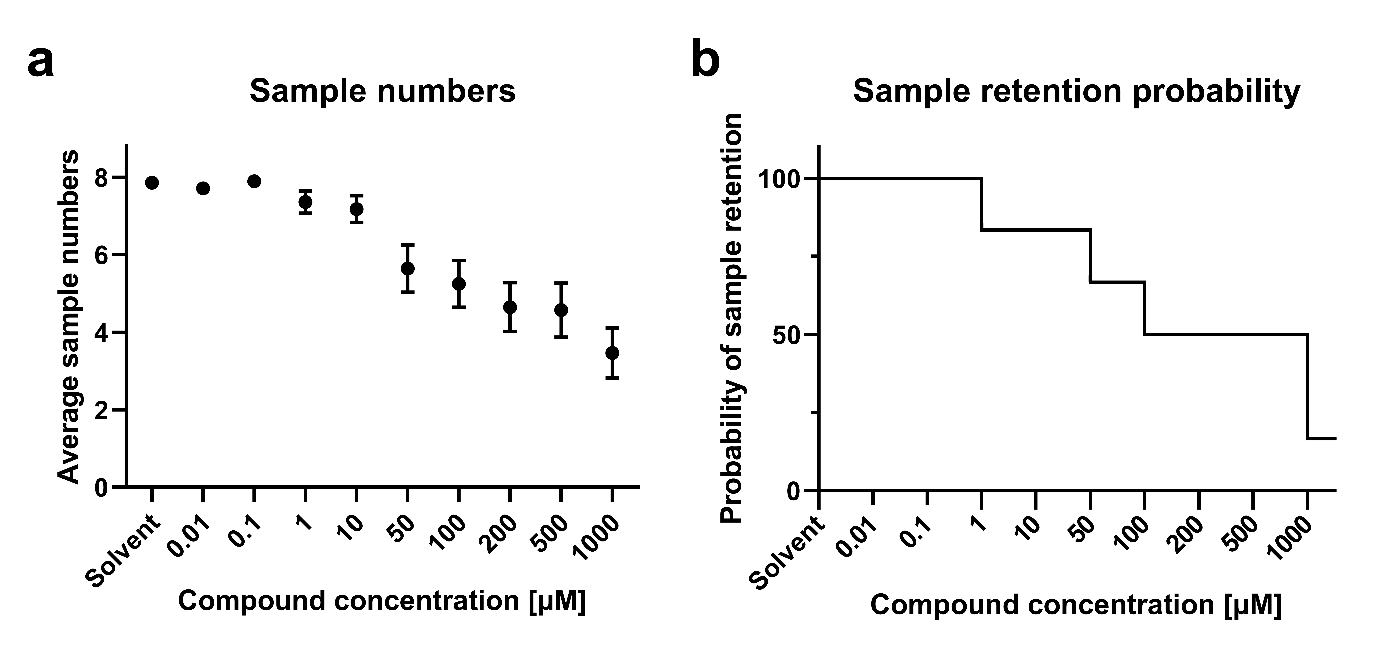


**Supplementary Figure 5. Compound concentration correlates with sample loss in the dose-response experiments**

Increasing concentrations of the compounds tested in our dose-response experiments result in a higher amount of sample loss. **a)** Sample numbers analyzed at the end of the dose-response experiments plotted against the compounds’ concentration. Depicted are the average sample numbers across all compounds, cell lines, and analysis types (i.e. viability and cell-type specific toxicity). Plotted is the mean +/- SEM, n = 28 organoids per concentration of the compounds. **b)** Emulated survival curve depicting the probability of sample retention at different compound concentrations. See methods section for details on data analysis and Table S3 for a detailed overview of sample numbers.

**Supplementary Tables**

**Table S1. Compound list including categories and the concentrations used in the primary screens**

| **Well** | **Compound** | **Category** | **Concentration in the primary screens [μM]** |
| --- | --- | --- | --- |
| A1 | 1-Ethyl-3-methylimidazolium diethylphosphate | Other | 101.0 |
| A2 | 1-Methyl-4-phenylpyridinium iodide | Neurotoxicant | 94.0 |
| A3 | 2-Ethylhexyl diphenyl phosphate (EHDP) | Flame retardant | 101.0 |
| A4 | 2-Ethylhexyl-2,3,4,5-tetrabromobenzoate (TBB) | Flame retardant | 100.0 |
| A5 | 2-Methoxyethanol | Neurotoxicant | 104.5 |
| A6 | 2,2',4,4',5-Pentabromodiphenyl ether (BDE-99) | Flame retardant | 100.0 |
| A7 | 2,2',4,4',5,5'-Hexabromodiphenyl ether (BDE-153) | Flame retardant | 100.0 |
| A8 | 2,2',4,4'-Tetrabromodiphenyl ether | Flame retardant | 100.0 |
| A9 | 2,3,7,8-Tetrachlorodibenzo-p-dioxin | Other | 0.5 |
| A10 | 3,3'-Iminodipropionitrile | Neurotoxicant | 102.0 |
| A11 | 3,3’,5,5’-Tetrabromobisphenol A | Flame retardant | 100.0 |
| A12 | 4-H-Cyclopenta(d,e,f)phenanthrene | Polycyclic aromatic hydrocarbon | 102.0 |
| B1 | 5-Fluorouracil | Drug | 103.0 |
| B2 | 6-Hydroxydopamine hydrochloride | Neurotoxicant | 100.5 |
| B3 | 6-Propyl-2-thiouracil | Drug | 102.5 |
| B4 | Acenaphthene | Polycyclic aromatic hydrocarbon | 102.0 |
| B5 | Acenaphthylene | Polycyclic aromatic hydrocarbon | 101.0 |
| B6 | Acetaminophen (4-hydroxyacetanilide) | Negative control | 103.0 |
| B7 | Acetic acid, manganese (2+) salt | Neurotoxicant | 98.0 |
| B8 | Acetylsalicylic acid | Negative control | 100.0 |
| B9 | Acrylamide | Neurotoxicant | 100.5 |
| B10 | Aldicarb | Pesticide | 99.4 |
| B11 | Amoxicillin | Drug | 85.5 |
| B12 | Anthracene | Polycyclic aromatic hydrocarbon | 99.5 |
| C1 | Auramine O | Other | 99.0 |
| C2 | Benz(a)anthracene | Polycyclic aromatic hydrocarbon | 100.5 |
| C3 | Benzo(a)pyrene | Polycyclic aromatic hydrocarbon | 101.5 |
| C4 | Benzo(b)fluoranthene | Polycyclic aromatic hydrocarbon | 99.2 |
| C5 | No treatment | No treatment | - |
| C6 | Benzo(k)fluoranthene | Polycyclic aromatic hydrocarbon | 99.0 |
| C7 | Benzo[g,h,i]perylene | Polycyclic aromatic hydrocarbon | 4.0 |
| C8 | No treatment | No treatment | - |
| C9 | Bis(2-ethylhexyl) 3,4,5,6-tetrabromophthalate (TBPH) | Flame retardant | 99.9 |
| C10 | Bis(tributyltin)oxide | Pesticide | 5.0 |
| C11 | Bisphenol A | Neurotoxicant | 99.1 |
| C12 | Bisphenol AF | Neurotoxicant | 99.5 |
| D1 | Bisphenol S | Neurotoxicant | 100.5 |
| D2 | Caffeine | Negative control | 100.0 |
| D3 | Captan | Pesticide | 99.5 |
| D4 | No treatment | No treatment | - |
| D5 | Carbaryl | Pesticide | 99.5 |
| D6 | DMSO | DMSO | 0.5% (v/v) |
| D7 | Chrysene | Polycyclic aromatic hydrocarbon | 100.0 |
| D8 | Colchicine | Drug | 99.0 |
| D9 | DMSO | DMSO | 0.5% (v/v) |
| D10 | Deltamethrin | Pesticide | 100.1 |
| D11 | Di(2-ethylhexyl) phthalate | Neurotoxicant | 100.0 |
| D12 | Diazepam | Drug | 101.5 |
| E1 | Dibenz(a,h)anthracene | Polycyclic aromatic hydrocarbon | 100.0 |
| E2 | Dibenz[a,c]anthracene | Polycyclic aromatic hydrocarbon | 99.5 |
| E3 | Dichlorodiphenyltrichloroethane (DDT) | Pesticide | 100.5 |
| E4 | Dieldrin | Pesticide | 107.7 |
| E5 | DMSO | DMSO | 0.5% (v/v) |
| E6 | Estradiol | Other | 100.0 |
| E7 | No treatment | No treatment | - |
| E8 | Fluorene | Polycyclic aromatic hydrocarbon | 100.0 |
| E9 | Heptachlor | Pesticide | 100.0 |
| E10 | Hexachlorophene | Pesticide | 100.0 |
| E11 | Hydroxyurea | Drug | 101.0 |
| E12 | Isodecyl diphenyl phosphate | Flame retardant | 100.5 |
| F1 | L-Ascorbic acid | Negative control | 100.0 |
| F2 | Lead (II) acetate trihydrate | Neurotoxicant | 100.5 |
| F3 | Lindane | Pesticide | 99.5 |
| F4 | Manganese, tricarbonyl[(1,2,3,4,5-.eta.)-1-methyl-2,4-cyclopentadien-1-yl]- | Neurotoxicant | 100.0 |
| F5 | Methyl mercuric (II) chloride | Neurotoxicant | 100.0 |
| F6 | n-Hexane | Neurotoxicant | 100.0 |
| F7 | Naphthalene | Polycyclic aromatic hydrocarbon | 99.5 |
| F8 | DMSO | DMSO | 0.5% (v/v) |
| F9 | Permethrin | Pesticide | 100.1 |
| F10 | Phenanthrene | Polycyclic aromatic hydrocarbon | 98.0 |
| F11 | Carbamic acid, butyl-, 3-iodo-2-propynyl ester | Pesticide | 99.5 |
| F12 | Benzo(e)pyrene | Polycyclic aromatic hydrocarbon | 99.5 |
| G1 | Phenol, isopropylated, phosphate (3:1) | Flame retardant | 99.8 |
| G2 | Pyrene | Polycyclic aromatic hydrocarbon | 100.5 |
| G3 | Rotenone | Pesticide | 101.0 |
| G4 | Saccharin Sodium Salt hydrate | Other | 101.0 |
| G5 | Tebuconazole | Pesticide | 101.0 |
| G6 | tert-Butylphenyl diphenyl phosphate | Flame retardant | 100.4 |
| G7 | Tetraethylthiuram disulfide | Drug | 100.0 |
| G8 | Thalidomide | Drug | 100.1 |
| G9 | Toluene | Neurotoxicant | 98.5 |
| G10 | Tricresyl phosphate | Flame retardant | 100.0 |
| G11 | Triphenyl phosphate | Flame retardant | 100.1 |
| G12 | Tris(2-chloroethyl) phosphate | Flame retardant | 100.5 |
| H1 | Valinomycin | Drug | 99.5 |
| H2 | Valproic acid sodium salt | Drug | 100.5 |
| H3 | tris(Chloropropyl) phosphate, TCPP | Flame retardant | 100.0 |
| H4 | Deltamethrin | Pesticide | 100.2 |
| H5 | Methyl mercuric (II) chloride | Neurotoxicant | 100.0 |
| H6 | Saccharin Sodium Salt hydrate | Other | 101.0 |
| H7 | Triphenyl phosphate | Flame retardant | 100.1 |
| H8 | Diethylstilbestrol | Drug | 99.4 |
| H9 | Chlorpyrifos (Dursban) | Pesticide | 100.0 |
| H10 | Firemaster 550 | Flame retardant | 101.3 |
| H11 | Berberine chloride | Other | 100.0 |
| H12 | D-Glucitol | Negative control | 101.0 |

**Table S2. Numbers of remaining samples after compound exposure for the primary cell type-specific toxicity screen**

|  |  | **Sample number** | |
| --- | --- | --- | --- |
| **Well** | **Compound** | **AMO line 1** | **AMO line 2** |
| A1 | 1-Ethyl-3-methylimidazolium diethylphosphate | 4 | 4 |
| A2 | 1-Methyl-4-phenylpyridinium iodide | 4 | 4 |
| A3 | 2-Ethylhexyl diphenyl phosphate (EHDP) | 4 | 4 |
| A4 | 2-Ethylhexyl-2,3,4,5-tetrabromobenzoate (TBB) | 4 | 4 |
| A5 | 2-Methoxyethanol | 4 | 4 |
| A6 | 2,2',4,4',5-Pentabromodiphenyl ether (BDE-99) | 4 | 4 |
| A7 | 2,2',4,4',5,5'-Hexabromodiphenyl ether (BDE-153) | 4 | 4 |
| A8 | 2,2',4,4'-Tetrabromodiphenyl ether | 4 | 4 |
| A9 | 2,3,7,8-Tetrachlorodibenzo-p-dioxin | 4 | 4 |
| A10 | 3,3'-Iminodipropionitrile | 4 | 3 |
| A11 | 3,3’,5,5’-Tetrabromobisphenol A | 4 | 3 |
| A12 | 4-H-Cyclopenta(d,e,f)phenanthrene | 2 | 4 |
| B1 | 5-Fluorouracil | 4 | 4 |
| B2 | 6-Hydroxydopamine hydrochloride | 4 | 4 |
| B3 | 6-Propyl-2-thiouracil | 4 | 4 |
| B4 | Acenaphthene | 4 | 4 |
| B5 | Acenaphthylene | 3 | 4 |
| B6 | Acetaminophen (4-hydroxyacetanilide) | 4 | 4 |
| B7 | Acetic acid, manganese (2+) salt | 4 | 4 |
| B8 | Acetylsalicylic acid | 4 | 4 |
| B9 | Acrylamide | 4 | 4 |
| B10 | Aldicarb | 4 | 3 |
| B11 | Amoxicillin | 4 | 4 |
| B12 | Anthracene | 2 | 3 |
| C1 | Auramine O | 3 | 4 |
| C2 | Benz(a)anthracene | 4 | 3 |
| C3 | Benzo(a)pyrene | 3 | 4 |
| C4 | Benzo(b)fluoranthene | 4 | 4 |
| C5 | No treatment | 4 | 4 |
| C6 | Benzo(k)fluoranthene | 4 | 4 |
| C7 | Benzo[g,h,i]perylene | 4 | 4 |
| C8 | No treatment | 4 | 4 |
| C9 | Bis(2-ethylhexyl) 3,4,5,6-tetrabromophthalate (TBPH) | 3 | 4 |
| C10 | Bis(tributyltin)oxide | 3 | 4 |
| C11 | Bisphenol A | 4 | 4 |
| C12 | Bisphenol AF | 0 | 1 |
| D1 | Bisphenol S | 4 | 4 |
| D2 | Caffeine | 4 | 4 |
| D3 | Captan | 4 | 4 |
| D4 | No treatment | 4 | 4 |
| D5 | Carbaryl | 4 | 4 |
| D6 | DMSO | 4 | 4 |
| D7 | Chrysene | 4 | 4 |
| D8 | Colchicine | 3 | 3 |
| D9 | DMSO | 4 | 4 |
| D10 | Deltamethrin | 4 | 4 |
| D11 | Di(2-ethylhexyl) phthalate | 4 | 4 |
| D12 | Diazepam | 2 | 3 |
| E1 | Dibenz(a,h)anthracene | 3 | 3 |
| E2 | Dibenz[a,c]anthracene | 4 | 4 |
| E3 | Dichlorodiphenyltrichloroethane (DDT) | 4 | 4 |
| E4 | Dieldrin | 4 | 4 |
| E5 | DMSO | 4 | 4 |
| E6 | Estradiol | 4 | 4 |
| E7 | No treatment | 4 | 4 |
| E8 | Fluorene | 4 | 4 |
| E9 | Heptachlor | 4 | 3 |
| E10 | Hexachlorophene | 0 | 1 |
| E11 | Hydroxyurea | 4 | 4 |
| E12 | Isodecyl diphenyl phosphate | 3 | 4 |
| F1 | L-Ascorbic acid | 3 | 4 |
| F2 | Lead (II) acetate trihydrate | 4 | 4 |
| F3 | Lindane | 4 | 4 |
| F4 | Manganese, tricarbonyl[(1,2,3,4,5-.eta.)-1-methyl-2,4-cyclopentadien-1-yl]- | 4 | 4 |
| F5 | Methyl mercuric (II) chloride | 4 | 4 |
| F6 | n-Hexane | 4 | 4 |
| F7 | Naphthalene | 4 | 3 |
| F8 | DMSO | 4 | 4 |
| F9 | Permethrin | 4 | 4 |
| F10 | Phenanthrene | 4 | 4 |
| F11 | Carbamic acid, butyl-, 3-iodo-2-propynyl ester | 4 | 4 |
| F12 | Benzo(e)pyrene | 2 | 4 |
| G1 | Phenol, isopropylated, phosphate (3:1) | 3 | 4 |
| G2 | Pyrene | 4 | 4 |
| G3 | Rotenone | 4 | 3 |
| G4 | Saccharin Sodium Salt hydrate | 4 | 4 |
| G5 | Tebuconazole | 3 | 3 |
| G6 | tert-Butylphenyl diphenyl phosphate | 4 | 4 |
| G7 | Tetraethylthiuram disulfide | 4 | 4 |
| G8 | Thalidomide | 4 | 4 |
| G9 | Toluene | 4 | 4 |
| G10 | Tricresyl phosphate | 4 | 4 |
| G11 | Triphenyl phosphate | 4 | 4 |
| G12 | Tris(2-chloroethyl) phosphate | 4 | 4 |
| H1 | Valinomycin | 4 | 4 |
| H2 | Valproic acid sodium salt | 4 | 4 |
| H3 | tris(Chloropropyl) phosphate, TCPP | 4 | 4 |
| H4 | Deltamethrin | 4 | 4 |
| H5 | Methyl mercuric (II) chloride | 4 | 4 |
| H6 | Saccharin Sodium Salt hydrate | 4 | 4 |
| H7 | Triphenyl phosphate | 4 | 4 |
| H8 | Diethylstilbestrol | 4 | 4 |
| H9 | Chlorpyrifos (Dursban) | 4 | 4 |
| H10 | Firemaster 550 | 4 | 4 |
| H11 | Berberine chloride | 4 | 4 |
| H12 | D-Glucitol | 4 | 4 |

**Table S3. Numbers of remaining samples after compound exposure for the dose-response experiments**

|  |  | | | **Compound concentration [μM]** | | | | | | | | | |
| --- | --- | --- | --- | --- | --- | --- | --- | --- | --- | --- | --- | --- | --- |
| **Compound** | | **Line** | **Readout** | **Control** | **0.01** | **0.1** | **1** | **10** | **50** | **100** | **200** | **500** | **1000** |
| Acetylsalicylic acid | | 1 | Viability | 8 | 8 | 8 | 8 | 8 | 8 | 8 | 8 | 8 | 8 |
|  |  |  | Staining | 8 | 8 | 8 | 8 | 8 | 5 | 6 | 7 | 7 | 6 |
|  |  | 2 | Viability | 8 | 8 | 8 | 8 | 8 | 8 | 8 | 8 | 8 | 8 |
|  |  |  | Staining | 6 | 7 | 7 | 5 | 7 | 5 | 6 | 4 | 6 | 7 |
| Bisphenol S | | 1 | Viability | 8 | 8 | 8 | 8 | 8 | 8 | 8 | 8 | 8 | 8 |
|  |  |  | Staining | 8 | 6 | 8 | 8 | 8 | 8 | 7 | 8 | 8 | 5 |
|  |  | 2 | Viability | 8 | 8 | 8 | 8 | 8 | 8 | 8 | 8 | 8 | 8 |
|  |  |  | Staining | 8 | 8 | 8 | 8 | 8 | 8 | 8 | 8 | 8 | 6 |
| Captan | | 1 | Viability | 8 | 8 | 8 | 8 | 8 | 8 | 6 | 6 | 8 | 5 |
|  |  |  | Staining | 7 | 7 | 8 | 8 | 8 | 8 | 7 | 6 | 8 | 2 |
|  |  | 2 | Viability | 8 | 8 | 8 | 8 | 8 | 8 | 8 | 7 | 8 | 3 |
|  |  |  | Staining | 7 | 8 | 8 | 8 | 7 | 7 | 4 | 4 | 8 | 3 |
| Berberine chloride | | 1 | Viability | 8 | 8 | 8 | 8 | 8 | 8 | 8 | 8 | 8 | 8 |
|  |  |  | Staining | 8 | 8 | 8 | 8 | 8 | 7 | 8 | 7 | 6 | 6 |
|  |  | 2 | Viability | 8 | 8 | 8 | 8 | 8 | 8 | 8 | 8 | 8 | 8 |
|  |  |  | Staining | 8 | 8 | 8 | 8 | 8 | 7 | 8 | 7 | 6 | 6 |
| Tetrabrom­bisphenol A | | 1 | Viability | 8 | 8 | 7 | 8 | 8 | 8 | 7 | 7 | 0 | 0 |
|  |  |  | Staining | 8 | 8 | 8 | 8 | 7 | 8 | 8 | 4 | 2 | 0 |
|  |  | 2 | Viability | 8 | 8 | 8 | 8 | 8 | 8 | 2 | 2 | 0 | 0 |
|  |  |  | Staining | 8 | 7 | 7 | 7 | 8 | 8 | 6 | 5 | 5 | 0 |
| Hexachloro­phene | | 1 | Viability | 8 | 8 | 8 | 8 | 7 | 0 | 0 | 0 | 0 | 0 |
|  |  |  | Staining | 8 | 6 | 8 | 2 | 4 | 0 | 0 | 0 | 0 | 0 |
|  |  | 2 | Viability | 8 | 8 | 8 | 7 | 0 | 0 | 0 | 0 | 0 | 0 |
|  |  |  | Staining | 8 | 7 | 8 | 3 | 3 | 0 | 0 | 0 | 0 | 0 |
| Rotenone | | 1 | Viability | 8 | 8 | 8 | 8 | 8 | 0 | 0 | 0 | 0 | 0 |
|  |  |  | Staining | 8 | 8 | 8 | 7 | 7 | 3 | 0 | 0 | 0 | 0 |
|  |  | 2 | Viability | 8 | 8 | 8 | 8 | 8 | 2 | 5 | 0 | 0 | 0 |
|  |  |  | Staining | 8 | 8 | 8 | 7 | 7 | 2 | 3 | 0 | 0 | 0 |
